# High return to sport rate and good patient-reported outcomes in recreational athletes following simple elbow dislocations

**DOI:** 10.1186/s13018-023-03914-2

**Published:** 2023-06-24

**Authors:** Philip-Christian Nolte, Melina Vorm Walde, Bryant P. Elrick, Paul-Alfred Grützner, Felix Porschke, Marc Schnetzke

**Affiliations:** 1 Department for Trauma and Orthopedic Surgery, BG Klinik Ludwigshafen, Ludwig-Guttmann-Strasse 13, 67071 Ludwigshafen, Germany; 2 Department of Orthopedics, University of Colorado School of Medicine, Aurora, USA; 3 German Joint Centre, ATOS Clinic Heidelberg, Heidelberg, Germany

**Keywords:** Elbow injury, Elbow instability, Return-to-sport, Elbow stabilization, UCL, LUCL

## Abstract

**Background:**

The purpose of this study was to investigate outcomes and return to sport metrics in recreational athletes who suffered simple elbow dislocations and were treated operatively or nonoperatively.

**Methods:**

The study included patients between the ages of 16 and 65 who were recreational athletes and had experienced a simple elbow dislocation, with at least 2 years having passed since the injury. Patient-reported outcomes including Mayo Elbow Performance Score (MEPS), Subjective Elbow Value (SEV), Oxford Elbow Score (OES) and Visual Analog Scale (VAS) were collected. Return to sport metrics were assessed.

**Results:**

A total of 44 patients (21 females, mean age 43.8 years [95% CI, 39.1–48.5]) who were recreational athletes before their injury completed follow-up at mean 7.6 years (95% CI, 6.7–8.5). There were 29 patients (65.9%) who were treated operatively. Mean MEPS was 93.3 (95% CI, 90.2–96.4), mean SEV was 94.9 (95% CI, 91.9–97.9) and mean OES was 43.3 (95% CI, 41.3–45.4). A total of 36 (81.8%) patients returned to their pre-injury sport. Mean time to return to sport was 21.7 (95% CI, 16.8–26.5) weeks. There was a significant difference in OES (*P* = .019) and SEV (*P* = .030) that favored the nonoperative group; however, no significant differences for MEPS, VAS, satisfaction, arc of motion and return to sport were present between groups. A total of five (11.4%) complications were observed and one (2.3%) required revision.

**Conclusions:**

Good outcomes and a high return to sport rate can be expected in recreational athletes following operative and nonoperative treatment of simple elbow dislocations. However, as many as one-in-five patients may not return to pre-injury sport.

## Background

Despite its inherent stability, the elbow is the second most commonly dislocated major joint in the general population with an incidence of 5.21 per 100.000 person-years [1, 2] and the fifth most commonly injured body part in young athletes [3]. Nearly, half (44.5%) of all elbow dislocations are sustained during sports activities, particularly during contact sports [2, 4]. Depending on the injury pattern, elbow dislocations are classified as either simple or complex. Simple dislocations are characterized by the absence of relevant osseous lesions, whereas complex dislocations involve fractures about the elbow [5].

Although the outcomes of operative and nonoperative treatment of simple elbow dislocations have been described in numerous studies [6–9], there is a paucity of literature investigating the return to sport rate, specifically for recreational athletes. Chang et al. [4] assessed the return to sport rate following simple elbow dislocations in professional National Football League (NFL) athletes. However, this population of professional athletes has unique physical constitution, motivation and access to medical treatment that likely does not reflect recreational athletes in the general population. Only one study thus far investigated patient-reported outcome scores (PROS) and return to sport rate in a cohort of presumably non-professional athletes [10].

**Fig. 1:**
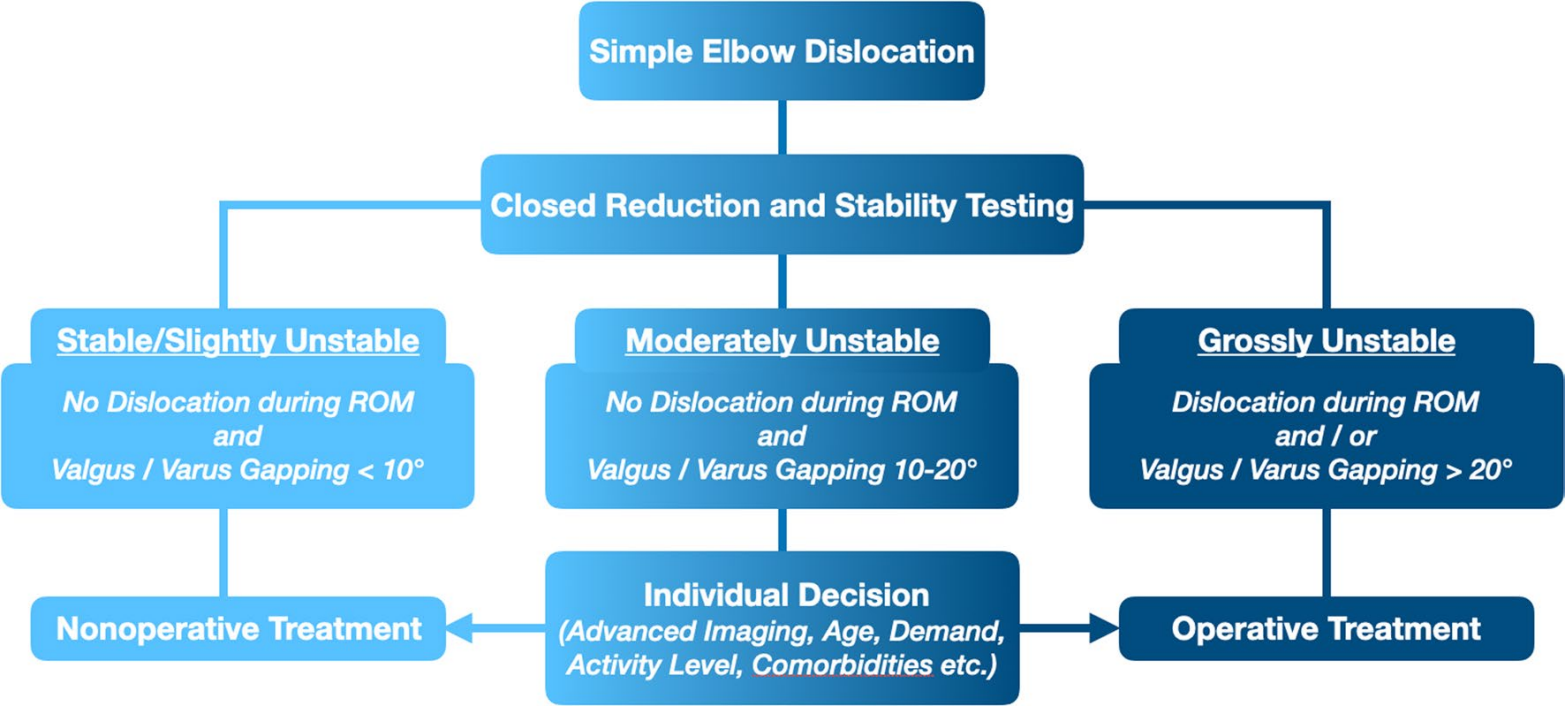
Decision tree on treatment following a simple elbow dislocation (on the basis of Schnetzke et al. [8, 9])

Therefore, the purpose of this study was to investigate outcomes and return to sport metrics in recreational athletes who suffered simple elbow dislocations and were treated operatively or nonoperatively. We hypothesized that good outcomes, few complications, and high return to sport rates would be observed for operatively as well as nonoperatively treated patients (Fig. 1).

## Methods

This retrospective, single-center study was performed at a level-I trauma center and approved by the local ethics committee. Patients between the age of 16–65 years with a minimum follow-up of 2 years who sustained an acute (< 3 weeks), simple elbow dislocation (no osseous injury except coronoid tip avulsions type I according to Regan and Morrey) from January 2008 to December 2019 and considered themselves non-professional athletes were included. Exclusion criteria were skeletally immature patients, a patient age < 16 and > 65 years at time of injury, concomitant injuries of the ipsilateral extremity, previous injuries to the elbow, open elbow dislocations and mental conditions such as dementia.

### Reduction and stability assessment

Immediately after reduction of the elbow joint under analgesia or anesthesia, joint range of motion was assessed, and stability testing was performed using fluoroscopy according to Schnetzke et al. [8, 9]. Elbows were considered stable/slightly unstable if full range of motion (full extension; e.g., 0°) was possible without subluxation or dislocation and if valgus/varus stress (full extension, 30° of flexion, pronation and supination) under fluoroscopic control did not reveal joint space gapping in excess of 10° [8, 9]. Elbows were considered moderately unstable if full range of motion was possible without subluxation or dislocation and if valgus/ varus stress revealed joint space gapping from 10° to 20° [8, 9]. If elbow dislocation occurred during range of motion testing or if valgus/varus stress demonstrated joint space gapping of more than 20°, elbows were considered grossly unstable [8, 9].

Following reduction and stability testing, the arm was placed in a long-arm cast with the elbow flexed at 90° and the forearm in neutral rotation, and standard radiographs were obtained. Of note, in some cases that had gross instability, the elbow was immobilized in > 90° of flexion to prevent re-dislocation.

### Nonoperative and operative treatment protocol

Stable or slightly unstable elbows were treated nonoperatively with long-arm cast immobilization until soft tissue swelling subsided; however, immobilization was carried out for no longer than a week before early active-assistive range of motion exercises began to avoid elbow stiffness. Starting from week three, active range of motion was initiated. Weight-bearing was prohibited for 6 weeks. Grossly unstable elbows were treated operatively. For moderately unstable elbows, decision-making for operative versus nonoperative management was performed individually based on further diagnostics such as magnetic resonance images, patient demand, activity level, age, and comorbidities (Fig. 1).

In the operating room, patients underwent clinical examination to determine the site of instability. The medial collateral ligament (MCL) and/or lateral collateral ligament (LCL) complexes and, if injured, the common flexor origin and/or the common extensor origin were repaired using metallic suture anchors (2.4 mm GII, DepuySynthes, Raynham, MA or 2.8 mm FASTak, Arthrex, Naples, FL) as indicated by stability testing. If necessary, the anterior capsule/coronoid tip avulsion was reattached with suture anchors as well. Our treatment algorithm included hinged external fixation if instability persisted despite repair of all injured structures. Notably, this was not necessary for any of the patients reported in this study. Following surgery, elbows were immobilized in a long-arm cast until soft-tissue swelling subsided. On postoperative day three, early active-assistive range of motion exercises begun, and starting from postoperative week three, active range of motion was performed. Weight-bearing was prohibited for 6 weeks.

### Demographic and surgery-related variables

Medical charts of eligible patients were queried and demographic variables such as age at injury, sex, hand dominance, treatment modality (operative vs. nonoperative) and type of procedure (repair of LCL and/or MCL and/or anterior capsule/ coronoid avulsion fracture) were collected.

### Clinical evaluation

Questionnaires were sent to patients via mail and included validated PROS such as the Mayo Elbow Performance Score (MEPS) [11], the Subjective Elbow Value (SEV) [12], the Oxford Elbow Score (OES) [13] and the Visual Analog Scale (VAS). Furthermore, elbow arc of motion (extension/flexion and pronation/supination) and patient satisfaction (1–6; 1 = most satisfied, 6 = least satisfied) were assessed. Patients were asked if they participated in sports before their elbow dislocation. If this was affirmed, the type and duration (hours per week) of sport were assessed. Patients were questioned regarding their ability to return to sport (full return to pre-injury sport [return to the same sport at the same level as before surgery], partial return to pre-injury sport [return to the same sport at a lower level as before surgery], no return to pre-injury sport but return to other sport [return to any type of sport at any level], no return to sport [no return to any type of sport]), and reasoning for non-return (limited range of motion, limited strength, fear of injury and elbow instability), if applicable. Time to return to sport (weeks) and duration (hours per week) at follow-up were assessed. Complications and revision surgeries were recorded. An arc of motion for elbow extension/flexion < 100° was considered a complication.

### Statistical analysis

Statistical analyses were performed using PRISM version 9.3.0 (GraphPad, San Diego, CA). Categorical variables are presented as numbers with frequencies, ordinal variables as median with range and continuous variables as mean with 95% confidence interval (95% CI). Normality of data was assessed using the Shapiro–Wilk test. The independent *t* test was used for univariate analyses of normally distributed data and the Mann–Whitney test for analyses of non-normally data. Bivariate data were analyzed using the Fisher exact test. Subgroup analyses were performed for nonoperatively and operatively treated patients. The level of significance was set at *P* < 0.05.

## Results

A total of 58 patients were eligible for inclusion and 44 patients completed minimum 2-year follow-up. Of the 44 included patients, 21 were female (47.7%) and 23 male (52.8%). The mean patient age was 43.8 years (95% CI, 39.1–48.5 years) and the mean follow-up was 7.6 years (95% CI, 6.7–8.5 years). Eighteen (40.9%) patients had comorbidities with hypertension being the most frequent (18.2%). There were only acute (< 3 weeks), simple elbow dislocations and all patients had an adequate trauma. The dominant arm was injured in 20 patients (45.5%), and 29 patients (65.9%) were treated operatively. Of the 29 patients that underwent surgery, eight patients had isolated LCL repair (27.6%), 11 patients had isolated MCL repair (39.9%) and ten patients underwent LCL and MCL repair (34.5%). Additionally, five patients underwent repair of the anterior capsule/ coronoid tip (17.2%) of which one patient (3.4%) had a coronoid tip avulsion Regan/Morrey type 1.

### Patient-reported outcomes and elbow arc of motion for the total cohort

The mean MEPS for the total cohort was 93.3 (95% CI, 90.2–96.4), the mean SEV was 94.9 (95% CI, 91.9–97.9), the mean OES was 43.3 (95% CI, 41.3–45.4), and the median VAS was 0 (range, 0–4). Median patient satisfaction at time of follow-up was 1 (range, 1–4). Mean arc of motion for extension/flexion of the elbow was 130.5° (95% CI, 125.5°–135.4°), and mean arc of motion for pronation/supination was 157.0° (95% CI, 153.5°–160.6°). Two patients (4.5%) had an arc of motion of less than 100° for extension/flexion.

### Return to sport analysis for the total cohort

All 44 patients were recreational athletes prior to their injury. Before injury, three (6.8%) patients participated in sports once a week, 29 (65.9%) patients participated in sports two to three times a week, and 12 (27.3%) patients participated in sports more than three times a week with a mean duration of 5.6 (95% CI, 4.6–6.6) hours per week. The most common sports performed pre-injury were biking (27.3%), weight training (25%), swimming (20.5%), running (15.9%), gymnastics (15.9%) and soccer (15.9%).

Although all patients returned to sporting activity of some kind, only 36 of 44 (81.8%) patients had full return to their pre-injury sport. There were six (13.6%) patients who had a partial return to pre-injury sport, one (2.3%) patient had no return to pre-injury sport but return to another sport, and one patient (2.3%) had no return to sport. Reasoning for the eight patients who did not fully return to pre-injury sport included limited range of motion (75%), pain (62.5%), limited strength (62.5%), fear of injury (50%) and elbow instability (37.5%). The mean time to return to sport was 21.7 weeks (95% CI 16.8–26.5) and patients participated in sports a mean of 5.0 (95% CI 3.9–6.1) hours per week at follow-up.

Patients who did not fully return to pre-injury sport had a significantly lower MEPS (94.9 [95% CI 91.7–98.1] vs. 86.3 [95% CI 76.3–96.2]; *P* = 0.010) and SEV (97.2 [95% CI 95.3–99.1] vs. 85.0 [95% CI 71.4–98.6]; *P* = 0.004), but no significant difference in OES (44.3 [95% CI 42.9–45.7] vs. 39.0 [95% CI 30.0–50.0]; *P* = 0.204) compared to those who did fully return to their pre-injury sport. Between those who fully returned to pre-injury sports and those who did not, there was no significant difference in age (43.0 years [95% CI 37.3–48.7] vs. 47.4 years [95% CI 41.2–53.5]; *P* = 0.958) or operative and nonoperative treatment (*P* = 0.695).

### Patient-reported outcomes, elbow arc of motion and return to sport for the subgroups

Demographic data for the operative and nonoperative groups is summarized in Table 1 and PROS and elbow arc of motion are demonstrated in Table 2. There were no statistically significant differences for the MEPS (*P* = 0.059), VAS (*P* = 0.498)_,_ satisfaction (*P* = 0.254), extension/ flexion (*P* = 0.277) and pronation/ supination (*P* = 0.282) arc of motion between groups; however, there were significantly higher OES (*P* = 0.019) and SEV (*P* = 0.030) in the group of patients that were treated nonoperatively. Return to sport parameters for both groups are demonstrated in Table 3. There were no statistically significant differences for sports participation pre-injury (*P* > 0.999), duration of sports pre-injury (*P* = 0.745), full return to sport (*P* = 0.695), time to return to sport (*P* = 0.349) and duration of sports at follow-up (*P* = 0.783).

**Table 1:** Demographic variables for the operative vs. nonoperative group

Demographic variable	Nonoperative (n = 15)	Operative (n = 29)	*P* value
Age at injury, years, mean (95% CI)	43.5 (36.6–50.4)	44.0 (37.5–50.5)	.552^c^
Male sex, n (%)	10 (52.6)	21 (53.8)	> .999^a^
Dominant arm injured, n (%)	4 (26.7)	15 (51.7)	.342^a^
Follow-up, years, mean (95% CI)	8.5 (7.4–9.6)	7.1 (5.9–8.3)	.128^b^

*CI* confidence interval

^a^Fisher’s exact test

^b^Unpaired *t* test

^c^Mann–Whitney test

**Table 2:** Outcomes and arc of motion for the operative and nonoperative group

	Nonoperative (n = 15)	Operative (n = 29)	*P* value
*Patient-reported outcome scores*			
MEPS, mean (95% CI)	97.7 (94.7–100.0)	91.0 (86.6–95.5)	.059
SEV, mean (95% CI)	98.9 (97.7–100.0)	92.7 (88.2–97.1)	**.030**
OES, mean (95% CI)	45.7 (43.4–47.9)	42.1 (39.2–45.1)	**.019**
Satisfaction, median (range)	1 (1–3)	1 (1–4)	.254
VAS rest, median (range)	0 (0–2)	0 (0–4)	.498
*Elbow arc of motion*			
Extension/flexion, mean (95% CI)	134.0 (127.4–140.6)	128.6 (121.7–135.5)	.277
Pronation/supination, mean (95% CI)	160.0 (160.0–160.0)	155.5 (150.2–160.9)	.282

*CI* confidence interval, *MEPS* Mayo elbow performance score, *VAS* visual analog scale, *OES* Oxford elbow score, *SEV* subjective elbow value. All tests for statistical significance performed with the Mann–Whitney test. Statistically significant values (*P* ≤ .05) are presented in bold

**Table 3:** Return to sport parameters for the operative vs. nonoperative group

Return to sport parameters	Nonoperative	Operative	*P* value
Duration of sport pre-injury, hours, mean (95% CI)	5.9 (3.9–8.0)	5.4 (4.3–6.6)	.745^b^
Full return to sport, n (%)	13 (86.7)	23 (79.3)	.695^a^
Time to return to sport, weeks, mean (95% CI)	19.5 (10.0–29.0)	22.8 (16.8–28.7)	.349^b^
Duration of sport at follow-up, hours, mean (95%CI)	5.1 (2.8–7.5)	4.9 (3.6–6.2)	.783^b^

*CI* confidence interval

^a^Fisher’s exact test

^b^Mann–Whitney test

### Complications and revisions

There were five (11.4%) complications. All of those occurred in the group that was treated operatively. One (2.3%) complication required revision surgery. There were no significant differences in complications between operatively and nonoperatively treated patients (*P* = 0.149). Complications and revision surgeries are listed in Table 4.

**Table 4:** Demographic variables and outcomes for the complications and revisions

Group	Age	Sex	RTS	Complication	Revision	MEPS	SEV	OES	SAT
Operative	52	Male	Partial return	Posttraumatic osteophytes with limited range of motion	Open arthrolysis + osteophyte removal	85	50	31	3
Operative	58	Male	Full return	Ulnar nerve injury	–	70	80	34	2
Operative	35	Female	Partial return	Painful scars	–	85	85	39	2
Operative	48	Male	Partial return	Arc of motion 80°	–	80	90	38	3
Operative	53	Male	No return	Arc of motion 70°	–	65	75	17	4

*MEPS* Mayo elbow performance score, *OES* Oxford elbow score, *RTS* return to sport, *SAT* satisfaction, *SEV* subjective elbow value

## Discussion

The most important finding of this study was that high return to sport rates and good PROS can be expected in recreational athletes following treatment of simple elbow dislocations. In the investigated cohort, all patients returned to sport although one in five patients (18.2%) did not return specifically to their pre-injury sport. The most common reason for failure to return to sport was limited range of motion. Analysis by treatment modality (e.g., operative vs. nonoperative) revealed that patients who were treated nonoperatively returned to sport earlier and were more likely to return to pre-injury sport; however, these differences were not statistically significant. It should be noted that individuals who underwent surgery displayed more instability during clinical and fluoroscopic (e.g., moderately and grossly unstable) testing. Therefore, it is possible that patients in the operative group had injuries that were more severe, making them harder to compare to patients who had nonoperative treatment. However, despite a presumably higher injury severity in the operative group, the groups demonstrated no significant differences regarding age, sex, hand dominance and follow-up time (Table 1). Additionally, this study demonstrates that the treatment protocol used resulted in good clinical outcomes which corroborates previous work on treatment of simple elbow dislocations [8, 9].

The presented findings are novel and can help physicians educate recreational athletes and manage expectations surrounding return to sport. Furthermore, these data will support future research in sports medicine regarding traumatic elbow dislocations and may be considered when establishing return to sport protocols for this injury.

There is a myriad of literature regarding return to sport in chronic elbow injuries, specifically medial collateral ligament injuries from overuse in throwing sports such a baseball [14–16]. However, despite almost half of all traumatic elbow dislocations resulting from sporting accidents, surprisingly little is known about return to sport rates following these injuries [2, 4]. Evidence in the literature is scarce and mostly limited to case reports involving professional athletes. In a case report, Uhl et al. [17] reported the case of a 21-year-old collegiate football player who had sustained a simple elbow dislocation during a game. The athlete underwent early functional rehabilitation using a hinged brace. He returned to practice 18-day post-injury followed by return to play at 3 weeks and remained healthy without re-injury for the remainder of the season. Verrall et al. [18] reported on three professional Australian Rules Football players who dislocated their elbows during a game and underwent immediate reduction with radiographs showing no signs of bony injuries. Patients were started on non-steroidal anti-inflammatory drugs and active mobilization exercises as well as physiotherapy commenced at 24-h and 48-h post-injury, respectively. The time to return to training (including tackling and body contact) for the three players was 5, 10 and 14 days, and time to competition (next game played) was 7, 13 and 21 days. At post-season assessment at 3 months, all players had full range of motion and no signs of instability.

In the largest cohort to date, Chang et al. [4] examined elbow dislocations in professional NFL athletes. A total of 62 elbow dislocations (0.17% of all injuries) were recorded of which 58 patients (93.5%) were treated nonoperatively and 4 (6.5%) underwent surgery. The treatment algorithm was not further specified, but the difference in distribution of operatively versus nonoperatively treated patients compared to our study is striking. Same season return to sport was achieved in 47 athletes (75.8%). Two underwent surgery and 45 were treated nonoperatively. The mean time to return to play was 26.0 days (range, 0.0–118 days) in athletes who returned during the same season. Athletes who were treated nonoperatively returned to play faster than athletes who underwent surgery (mean 25.1 days [range, 0.0–118 days] vs. mean 46.5 days [range, 29–64 days]) which corroborates the findings of our study that return to sport may be faster with nonoperative treatment. Interestingly, return to play time differed depending on player position with offensive players returning faster compared to defensive players (mean 24.1 days [range, 2.0–59] vs. 25.8 days [range, 3.0–118]; *P* = 0.42).

As mentioned above, in the study of Chang et al. [4], only 6.5% of patients were treated operatively. Similarly, Anakwe et al. [7] investigated 110 patients following simple elbow dislocations, and only 4% were treated operatively. In the present cohort, almost 66% of patients were treated operatively. While the gold standard for the treatment of simple elbow dislocations remains nonoperative, results are not always favorable [9]. As an example, Anakwe et al. found that 16% of patients had objective (8%) or subjective (8%) instability following nonoperative treatment [7], and up to one third of patients report a mild to moderate decrease in range of motion [19].

Treatment choice is commonly based upon stability and joint congruency following closed reduction. However, how instability is assessed may vary (clinical, fluoroscopic and sonographic) and result in different proportions of patients being treated operatively. When fluoroscopy is used to assess instability, as was the case in the present study, other investigations have reported similar high percentages of operatively treated patients: Beirer et al. [20] treated 50% of their patients operatively and found no significant differences in patient-reported outcomes compared to the nonoperative group. We assume that when the diagnosis of instability is solely based on clinical examination, there may be a few patients that remain underdiagnosed and would do better with surgery. This supports the notion that a simple elbow dislocation is not always “simple” but can be a complex, soft-tissue injury requiring surgery [21].

Chang et al. [4] did not investigate the return to sport rate or return to sport time for athletes that were not able to return during the same season, therefore potentially underestimating these values. In the author’s opinion, their results are not applicable to the general population since they investigated only professional athletes and are difficult to extrapolate and compare to the findings of the current study. Professional athletes have unique access to medical treatment and intense physiotherapy. Also, it is likely that they are highly motivated to return to sport as quickly as possible to avoid affecting their career negatively. Furthermore, it is the authors’ opinion that professional athletes are in resilient physical condition when the injury occurs, which potentially expedites their recovery. For instance, the return to sport time of just 24 days following nonoperative treatment is not to be expected in a non-professional athlete. Although Chang et al. [4] only included athletes that were able to return to the same season, their return to sport time was much quicker (26.0 days) than what we found in a general population (21.7 weeks). This difference of more than 120 days is even more striking, when considering that the cohort of Chang et al. [4] was solely comprised professional contact athletes (NFL) as opposed to non-contact, recreational athletes in our cohort.

Recently, Geyer et al. [10] published their data on outcomes and return to sports following operative and nonoperative treatment of simple elbow dislocations. Although it was not specifically stated whether professional athletes were included in this cohort when comparing the level of sports in the study of Geyer et al., it is likely that the majority of patients were non-professional athletes (only seven of 44 patients played sports daily) making their cohort comparable to ours.

The authors investigated 44 patients of whom 37 performed sports before the injury and demonstrated return to sports for all 37 patients regardless of the treatment received. Of note, two of 37 patients (5.4%) performed sports less often, and six of 37 patients (16.2%) changed sports following simple elbow dislocation which equates to the “partial return to pre-injury sport” group and the “no return to pre-injury sport but return to other sport” group of the present study, respectively. The findings of Geyer et al. therefore corroborate the “full return to pre-injury sports” rate of 81.8% that we have found.

Interestingly, Geyer et al. reported a significantly faster return to sport time for operatively treated (mean 3 ± 4.9 months) compared to nonoperatively treated patients (mean 6 ± 20.4 months; *P* = 0.036). This may be due to the types of sports that patients performed with more patients who participated in non-upper extremity sports and thus potentially quicker return to sport; however, the types of sports were relatively similar (biking, fitness/weight training, running). Another reason may have been that both studies were retrospective, and therefore, recall bias may have been problematic after a follow-up of more than 5 years in the study of Geyer et al. and more than 7 years in the present study. Additionally, we evaluated the time to “full return to pre-injury sport,” whereas Geyer et al. investigated the time to return to any type of sport. Since Geyer et al. included eight patients that performed sports less often or/and changed sports following injury in their analysis, return to sport time may have been quicker compared to our cohort. In contrast to the findings of the present study, Geyer et al. had a higher rate of complications (9.5%) in the group of nonoperatively treated patients. Among the potential reasons may be the difference in indicating surgical treatment when compared to our study. Fluoroscopic evaluation of valgus and varus gapping was not performed, potentially underdiagnosing patients when surgery would have been indicated. The MEPS was excellent for both operative (98.7 ± 3.3) and nonoperative treatment (97.3 ± 6.8) without significant differences between groups which is similar to the nonoperative group in our study but higher for the operative group. However, it is questionable whether a difference of 7.7 points in the operative group is also clinically significant.

### Limitations

This study had several limitations. First, this was a retrospective study which made it susceptible to bias such as loss of data. Second, we did not specifically investigate the return to sport rate by type of sport performed; however, most of them can be considered low impact. Third, despite using validated PROs, no in-person examination was performed, and clinical significance values are not yet established for elbow dislocations. Finally, the subgroups (operative vs. nonoperative) were heterogeneous. This is because the operative group had a higher burden of injury (higher grade of instability) making it difficult to draw conclusions based on this data whether one treatment is superior to the other in terms of PROS and return to sport.

## Conclusions

Good outcomes and a high return to sport rate can be expected in recreational athletes following operative and nonoperative treatment of simple elbow dislocations. However, as many as one-in-five patients may not return to pre-injury sport.

## Data Availability

Data is available from the authors upon reasonable request.
